# Surgical Treatment of Meningioma with Situs Inversus Totalis Assisted by 3D Technology: A Case Report

**DOI:** 10.2174/0115734056459133251211072449

**Published:** 2026-01-30

**Authors:** Hao-Dong Luo, Yang Liu, Jian-Feng Xu

**Affiliations:** 1 Department of Neurosurgery, Affiliated Hospital of Southwest Medical University, Luzhou City, 646000, China; 2 Department of Neurosurgery, The Third Hospital of Mianyang, Mianyang, 621000, China; 3 Department of Neurosurgery, Sichuan Mental Health Center, Sichuan, 621000, China; 4 Department of Neurosurgery, The Affiliated Mianyang Hospital of Chongqing Medical University, Mianyang, 621000, China

**Keywords:** Situs inversus totalis, Meningioma, 3D printing, Situs inversus totalis, Congenital anomaly, Symmetrical facial movements

## Abstract

**Background:**

Meningiomas (MGM) are common intracranial tumors, while complete situs inversus totalis (SIT) is an uncommon congenital anomaly. However, there are few documented cases of complete situs inversus coexisting with brain tumors, and particularly, there have been no reports on the relationship between surgically treated MGM and complete situs inversus.

**Case Presentation:**

A 52-year-old female, presenting with a 7-month headache history, worsening over the past 10 days, with new-onset left lower limb weakness. She reported difficulty lifting the left leg, dragging during ambulation, and a “stepping on cotton” sensation. No significant past medical history.

**Conclusion:**

This case highlights that the surgical approach must be determined based on the precise tumor-to-brain anatomy provided by 3D printing technology, while also accounting for the patient’s complete situs inversus and dominant hand.

## 1 INTRODUCTION

MGM originates in the meninges as well as commonly affects individuals aged 40–60 years, with a female predominance (male-to-female ratio 2:3 to 1:2). Their incidence ranges from 1/10,000 to 1–8/100,000, representing around 37.6% of all primary intracranial tumors [1]. WHO classifies MGM into three grades: Grade I (benign, >80% 10-year net survival), Grade II (atypical, ~5–7%, 10-year survival >60%), and Grade III (anaplastic, 1–3%, 10-year survival 15%) [2]. Surgery stands as the primary treatment, particularly for Grade I MGM, with gross total resection as the goal [3].

SIT, a rare congenital malformation occurring in 1 out of every 8,000 to 25,000 individuals, is marked by the complete transposition of thoracic as well as abdominal organs. It is also referred to as “mirror image anatomy” [4, 5]. SIT associated with tumors is extremely rare, with fewer than 100 cases reported since Allen's first description in 1936 [6]. There have been no prior reports of MGM coexisting with SIT.

## 2 CASE PRESENTATION

A 52-year-old female was admitted on January 23, 2025, presenting with a 7-month history of headaches, worsening over the past 10 days, with new-onset left lower limb weakness. She reported difficulty lifting the left leg, dragging during ambulation, and a “stepping on cotton” sensation. No significant past medical history.

**Fig. (1):**
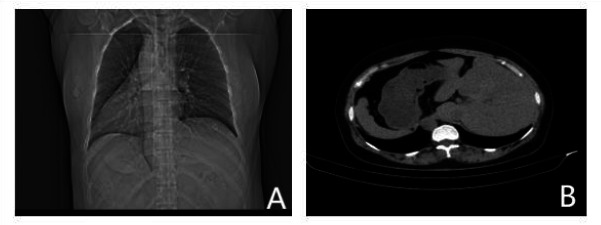
Shows that CT confirmed the patient’s SIT: (**A**) shows the right side of the heart on a chest CT scan; (**B**) shows the left and right internal organs inverted.

**Fig. (3):**
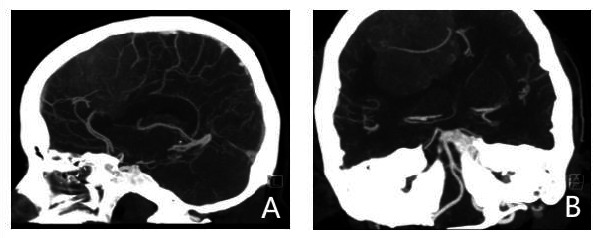
Three-dimensional imaging of intracranial arteries using CT: (**A**) Sagittal view; (**B**) Coronal view.

Neurological examination revealed a conscious patient with isocoric, round pupils and intact light reflexes. Extraocular movements were full; no nystagmus or gaze deviation was observed. Symmetrical facial movements and nasolabial folds. Left-handedness. Muscle strength: upper limbs and right lower limb 5/5; left lower limb paresis evident with a positive weakness test. After admission, a cranial MRI and chest CT scan revealed a right frontal mass and SIT. Figure **1** shows that CT confirmed the patient’s SIT, with Fig. (**1A**) displaying the heart on the right side *via* chest CT, and Fig. (**1B**) revealing an inversion of the left-sided organs with the right-sided organs Fig. (**1B**). A supplementary physical examination revealed a sharp pulsation located at the fifth intercostal space on the midline of the right clavicle. Fig. (**2**): Cranial magnetic resonance imaging (MRI) indicates a right frontal lobe mass measuring approximately 7.5 cm × 5.6 cm × 6.8 cm with clear boundaries, as well as a shift of approximately 1.7 cm to the left of the brain midline Fig. (**2**). Based on the MRI, it is preliminarily considered that the patient is likely to have a meningioma, and the tumor is located in a functional area. The patient had SIT. Further refinement of the three-dimensional imaging of the intracranial artery CT revealed a mass-like space-occupying lesion within the right frontal lobe, compression of the adjacent lateral ventricle, leftward displacement of the brain midline structure, compression and displacement of adjacent anterior cerebral artery, as well as a large branch of the right anterior cerebral artery as blood supply vessel, running under the lower part of the tumor (Fig. **3**).

## 3 PREOPERATIVE PLANNING

Based on MRI measurements, we identified this lesion as a giant meningioma. Giant MGMs, typically defined as those with a diameter ≥6 cm, are exceptionally rare. Their characteristics often pose significant challenges for surgeons attempting gross total resection [7]. Related studies show that giant MGMs have a higher overall risk of complications than normal MGMs. The complication rate in a series of giant meningioma surgeries reported by Haeren *et al*. ranged from 57% to 64% [8]. In a study by Teama *et al*. of 48 patients undergoing surgery for giant meningioma, 14 patients (29%) developed new cranial nerve deficits. Among the group, only 8 patients recovered completely and returned to their normal status within 3 months, while the remaining 6 patients had persistent cranial nerve deficits [9]. In a study looking at 66 patients diagnosed with giant meningioma, Yin *et al*. indicated a higher incidence of neurological complications than standard MGM, and hemiparesis was the main postoperative neurological deficit [10].

The intricacy and the level of accuracy required in neurosurgical operations, coupled with the high level of anatomical expertise demanded by the practice, therefore render surgical practitioners to endure a lengthy learning curve [11]. Traditional two-dimensional (2D) imaging modalities, such as X-ray, CT, and MRI, fail to provide a complete and tangible picture of the patient's pathological anatomy [12, 13]. These shortcomings of conventional imaging and the clinical demands made by neurosurgeons are the very factors that have led to the adoption of 3D printing technology in skull base surgery. The development of this technology allows surgeons to create accurate anatomical models during preoperative planning, thus improving the stereoscopic knowledge required for such complex interventions and providing intuitive knowledge and a complete analysis of complex tumor anatomy [14].

Research shows that assisted tumor surgery using 3D imaging is better for visualizing meningiomas, sellar-region tumors, and cerebellopontine angle tumors, thereby minimizing surgical risks [15]. The applications highlight the importance of 3D printing in minimizing surgical complications across different central nervous system tumor types. Quantitative evidence of such benefits has also recently been offered through research. For instance, in a study by Dho Y-S *et al*., the preoperative 3D-printed tumor models led to modifications of the originally planned resection extent in 18.8% of cases (12 out of 64 patients). In most cases, the surgical plan was adjusted to achieve more precise tumor resection [16]. Additionally, compared with MRI alone, the 3D models markedly enhanced critical surgical planning parameters, enabling more precise determination of optimal head positioning and craniotomy design [17].

**Fig. (4):**
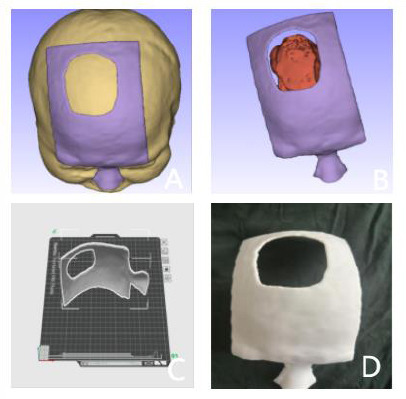
(**A**) 3D tumor model constructed by 3D Slicer; (**B**) Optimization of model details; (**C**) Manufacturing process of the printed model; (**D**) Final 3D printed product.

Literature suggests that in SIT, cerebral lateralization (*e.g*., language dominance) may be reversed [18]. Given the tumor's right frontal location in this case, we documented that the patient was left-handed. Electroencephalography (EEG) revealed asymmetry of bilateral alpha waves. Based on these findings and the previously completed CTA of the intracranial arteries, we proceeded with the Wada test. The patient was positioned supine. The right groin area was shaved, disinfected, and draped with sterile towels. Under local anesthesia, a needle was inserted into the femoral artery *via* a percutaneous puncture. A catheter was advanced under C-arm guidance to the right internal carotid artery. A solution of 100 mg of amobarbital sodium dissolved in 10 ml of normal saline was injected over approximately 5 seconds. Immediately after the injection, the patient was instructed to count backwards from 100 (100, 99, 98...). Speech arrest occurred 50 seconds after the drug administration. This finding again indicated right-hemisphere dominance for language, consistent with the literature. Therefore, to protect the cerebral parenchyma, vascular structures, and the patient’s language function, we implemented additional precautionary measures by employing 3D printing technology (Fig. **4**).

## 4 INTRAOPERATIVE AND POSTOPERATIVE FINDINGS

Under the guidance of fluorescence angiography (skin test negative), a right frontotemporal horseshoe-shaped incision was made, crossing the midline to expose the cerebral falx. The scalp was incised, and the bone flap was elevated, revealing tense dura mater. The dura was tacked up and then opened in a curvilinear fashion toward the cerebral falx. Intracranial pressure was also high, and there was gross protrusion of brain tissue. The lesion did not only affect the cerebral cortex; it was also characterized by significant adhesion to the meninges. The lesion was carefully dissected and resected under microscopic guidance using Fluorescence angiography. It was hard, solid, well-delimited, highly vascularized, and the surrounding brain tissue was edematous. The lesion compressed the corpus callosum inferiorly, with its base attached to the cerebral falx. The resected lesion measured approximately 6 × 5 × 6 cm. Following resection, a significant decrease in intracranial pressure was observed, and cerebral pulsation was satisfactory.

Postoperative pathological biopsy of resected lesion tissue is described in Fig. (**5**): pathologic diagnosis, Meningioma (WHO Grade 1). Immunohistochemistry: EMA (focal +), PR (+), S-100 (focal +), GFAP (-), SOX-10 (-), STAT6 (-), CD34 (vascular +), Ki-67 (~5%)

## 5 FOLLOW-UP AND OUTCOME

### 5.1 From Surgery to Discharge

**Fig. (6):**
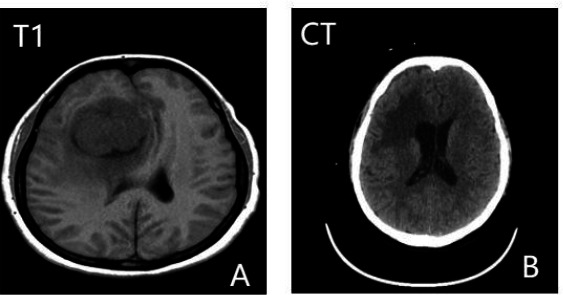
(**A**) Axial view of the preoperative T1-weighted MRI; (**B**) Axial view of the CT scan performed at discharge.

On the 10th postoperative day, the patient’s symptoms had significantly alleviated. At discharge, physical examination revealed the patient to be alert and fluent. Bilateral pupils were equal and round, with a diameter of 2.5 mm. Muscle strength was graded 5/5 in all four limbs, with no notable abnormalities in muscle tone. Pathological reflexes were absent, meningeal signs were negative, and there were no reports of a sensation of walking on cotton or clinical signs of ataxia. Follow-up imaging at discharge showed marked improvement compared with the preoperative studies (Fig. **6**).

### 5.2 Two Months Postoperatively

**Fig. (7):**
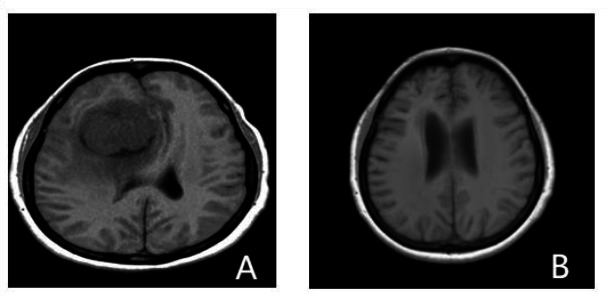
(**A**) Axial view of the preoperative T1-weighted MRI; (**B**) Axial view of the MRI performed at the two-month postoperative follow-up.

Two months post-surgery, the patient’s MRI showed significant improvement compared to the preoperative study (Fig. **7**). The patient remained asymptomatic with no reported discomfort or abnormal neurological signs.

### 5.3 Eight Months Postoperatively

**Fig. (8):**
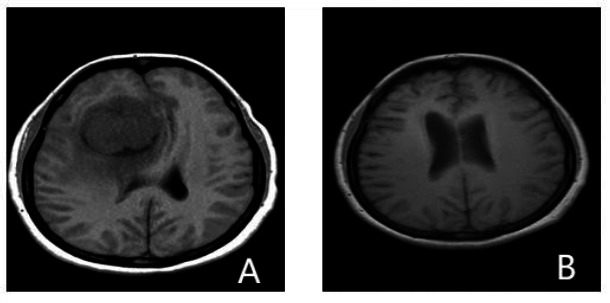
(**A**) Axial view of the preoperative T1-weighted MRI; (**B**) Axial view of the MRI performed at the eight-month postoperative follow-up.

At the 8-month postoperative follow-up, MRI revealed significant improvement compared to the preoperative study (Fig. **8**). The patient remained asymptomatic and exhibited no neurological deficits.

## 6 DISCUSSION

The etiology of SIT remains unclear but is believed to involve ciliary dysfunction and genetic mutations [19]. Risk factors include family history, maternal diabetes, paternal smoking, and low socioeconomic status [20]. Boskovski M’s study showed that simple SIT is often associated with familial genetic factors, changes in chromosome structure and number, as well as fetal ciliary motility disorders. Although many factors influence SIT, the overall incidence rate is not high, and SIT patients can function at a high level like people with normal organ positioning, and it typically exerts no significant impact on their quality of life or longevity [21]. While SIT rarely impacts life expectancy, it complicates surgical planning due to reversed anatomy. To date, open surgical treatment of intracranial tumors in SIT patients has not been previously reported. MGM represents the predominant form of primary intracranial tumors, characterized by high incidence, slow growth, and genetic variation [22]. Severe symptoms induced by MGM are usually attributed to secondary effects stemming from alterations in peripheral tissue pressure or hemorrhage, primarily manifesting as headaches, dizziness, vomiting, behavioral abnormalities, epilepsy, *etc* [23]. Surgery remains the most effective treatment [24]. In SIT patients, surgical challenges are compounded by organ transposition and potential vascular anomalies [25]. When treating tumors with SIT combined with surgery, some scholars have suggested that factors such as arterial and venous contact with the tumor, distant metastasis, cardiopulmonary insufficiency, and hepatic and renal insufficiency must be taken into consideration [26]. 3D-Slicer is a software platform jointly developed by Harvard University and the Massachusetts Institute of Technology (MIT) for medical image informatics, image processing, and three-dimensional visualization. The primary functions of 3D Slicer include image visualization and image-guided therapy, enabling image analysis, real-time image editing, and registration. It applies to all organs and tissues of the human body [27]. 3D Slicer allows integration of CT and MRI datasets to generate accurate 3D reconstructions, facilitating surgical planning, intraoperative navigation, and deepening comprehension of intricate anatomical interconnections [28]. After surgical treatment of this case, we summarize as follows: (1) The tumor compression symptoms are obvious and have already affected limb muscle strength and sensation, so surgery is the first choice. (2) Given the right frontal lobe mass identified in the patient and considering literature suggesting that individuals with SIT may have reversed cerebral language dominance compared to the typical population, our preoperative planning included a Wada test to elucidate lateralization further. The test confirmed right-hemispheric language dominance. Following a multidisciplinary discussion, our department decided to employ 3D printing technology to better visualize the spatial relationship between the tumor and adjacent structures and to optimally plan the surgical incision. (3) In this case, 3D printing was pivotal for preoperative design, optimizing the trajectory and reducing potential neurovascular injury. (4) Reverse anatomical awareness and team coordination were essential to surgical success. (5) Long-term outcomes of meningioma surgery in SIT patients warrant further study. Although SIT patients are rare, this case is worth taking as a reference. (6) Furthermore, the long-term prognosis of such rare cases requires validation through multi-center, large-sample follow-up studies, which is essential for guiding future research directions.

## 7 LIMITATIONS OF THE STUDY

Although this case showed a successful surgical outcome, there are still some limitations: single case samples, lack of control cases, and short follow-up time.while this case highlights a promising and meticulously planned surgical strategy, its implications should be considered within the context of these limitations. Future multi-center studies with larger sample sizes and longer follow-up durations are essential to validate these preliminary findings, establish standardized protocols, and better define the role of 3D printing in managing such rare and complex neurosurgical conditions.

## CONCLUSION

To conclude, surgery is the primary treatment for patients with symptomatic meningioma. Nevertheless, there is still a high likelihood of occurrence of neurological complications as a result of the procedure, especially in patients who have giant MGM in the dominant hemisphere, whereby they are prone to the highest number of deficits. This case report presents an infrequent case of giant meningioma in a patient with SIT who was able to undergo surgical excision without any complications after the removal of the tumor. When the patient was admitted, a cranial MRI was performed promptly, revealing the lesion. Routine thoracic imaging then revealed the rare SIT disorder. Based on the literature, a preoperative assessment of hemispheric dominance was performed, and it was found that the tumor was in the language-dominant hemisphere. Given that the lesion’s diameter exceeds 6 cm, classifying it as a giant meningioma, 3DP was used to precisely depict the relationship between the mass and adjacent structures and to accurately plan the craniotomy. The meningioma was successfully excised, resulting in significant symptom improvement. Subsequent investigations did not show any neurological impairment. This case report is intended to highlight that surgical intervention remains the preferred therapeutic approach for such patients. The adoption of 3DP technology would help reduce unnecessary neural injury, intraoperative blood loss, and tumor exposure, as well as ease patients' symptoms, thus leading to a positive surgical outcome. The case is intended to provide clinicians who encounter such a complex presentation with useful information in the future. Furthermore, the accumulation of analogous cases holds the potential to advance our understanding of MGM in the context of SIT and inform the development of more comprehensive and tailored therapeutic strategies.

## Figures and Tables

**Fig. (2):**
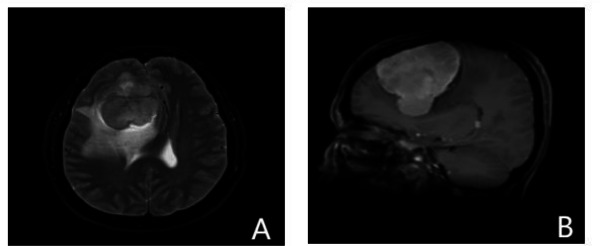
Cranial MRI: (**A**): horizontal view;(**B**): sagittal view.

**Fig. (5A, B):**
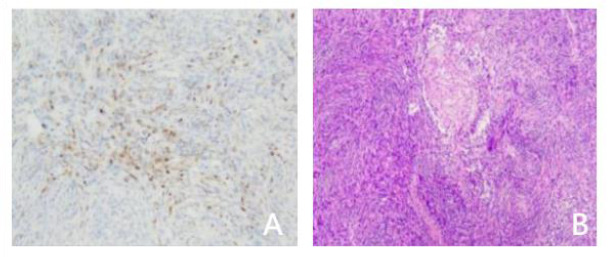
Pathological biopsy of the diseased tissue.

## Data Availability

Not applicable.

## LIST OF ABBREVIATIONS

MGM: Meningiomas
SIT: Situs inversus totalis
3DP: Three-dimensional printing
WHO: World Health Organization
MRI: Cranial magnetic resonance imaging
EEG: Electroencephalography
MIT: Massachusetts Institute of Technology

## STANDARDS OF REPORTING

CARE guidelines were followed.
